# Downregulation of circulating miR-320a and target gene prediction in patients with diabetic retinopathy

**DOI:** 10.1186/s13104-020-05001-9

**Published:** 2020-03-16

**Authors:** Marcelle SanJuan Ganem Prado, Mirthz Lemos de Jesus, Thaline Cunha de Goes, Lucilla Silva Oliveira Mendonça, Carla Martins Kaneto

**Affiliations:** 1 Department of Health Science, Universidade Estadual de Santa Cruz, Ilhéus, BA Brazil; 2 Department of Biological Science, Universidade Estadual de Santa Cruz, Rodovia Jorge Amado, Km16, Ilhéus, BA 45662-900 Brazil

**Keywords:** MicroRNA, Diabetic Retinopathy, Circulating microRNAs, Biomarker

## Abstract

**Objective:**

To evaluate the expression of a set of miRNAs to identify differentially expressed miRNAs that might be considered reliable biomarkers on Diabetic Retinopathy (DR) blood samples.

**Results:**

Expression levels of MiR-320a, MiR-342-3p, MiR-155, MiR-99a, MiR-29a and MiR-27b were analyzed in 60 healthy controls, 48 Diabetes Melitus (DM) without DR patients and 62 DR patients by qRT-PCR. MiR-320a was shown to be downregulated in the plasma of DR patients compared with DM patients without DR and healthy subjects. Target genes were predicted using miRWalk3.0, miR targeting data and target gene interaction data were imported to Cytoscape to visualize and merge networks and top ranked predicted genes were run through Ontology Genes to perform enrichment analysis on gene sets and classification system to identify biological processes and reactome pathways associated with DR. Highly scored target genes of miR-320a were categorized for various biological processes, including negative regulation of cell aging, negative regulation of cellular protein metabolic process and regulation of cellular response to stress that are critical to the development of DR. Our findings suggest that MiR-320a may have a role in the pathogenesis of DR and may represent novel biomarkers for this disease.

## Introduction

Diabetic retinopathy (DR), a leading cause of acquired vision impairment and blindness among working-age adults, is a frequent microvascular complication of diabetes melitus (DM) [1–4]. Traditional risk factors for DR include longer diabetes duration, dyslipidemia, high blood pressure and poor blood glucose control [5], but epidemiological data suggest that differential genetic susceptibility may be related to this chronic complication [6] and epigenetics mechanisms, such as non-coding RNAs, are supposed to mediate the interplay between genetic and environmental factors.

Predicting the clinical course of the disease is often difficult for many DM patients highlighting the necessity of development of sensitive, specific and widely available clinical laboratory-based monitoring testes for this condition and the importance of improving our knowledge of the pathogenesis of DR [7]. A biomarker would allow potential early treatment of DM patients who are at high risk of developing DR, could help to predict the progression of DR to vision threatening DR or the identification of low risk people, but until date, no ideal biomarkers for identifying or predicting DR have been determined.

MicroRNAs are single stranded, short length (21 to 23 nucleotides) non-coding RNA molecules that regulate post-transcriptional gene expression by binding to the complementary sites of targets mRNA and have important functions in gene regulatory networks [8–10]. In the past few years, scientists have found that miRNA can be rapidly released from tissues into the circulation with the development of a pathology and aberrant expression of circulating miRNAs has been detected in a wide range of pathological conditions including cancer [11, 12], diabetes [13, 14], cardiovascular [15–18] and neurodegenerative [19] diseases. They were recently demonstrated to be transported between cells as well as circulate in body fluids [20, 21] and these findings have inspired a great using extracellular circulating miRNAs as non-invasive biomarkers for molecular diagnostics, disease stratification and prognostics.

Recent studies have detected miRNAs in the blood or vitreous humor of DR patients, suggesting that miRNA may be involved in DR and that some miRNAs may be biomarkers for DR [22–26], but many of them focused on miRNAs expressed in cells or animal models, so the global miRNA pattern in the sera of plasma samples of DR patients has not been determined.

Herein, we investigated expression levels of miR-320a, miR-342-3p, miR-155, miR-99a, miR-29a and miR-27b in healthy people, DM without DR patients and DR patients by qRT-PCR aiming to find specific miRNAs that could serve as reliable and reproducible biomarkers for DR. This candidate microRNAs were selected from a review of previously published studies and were also chosen based on using prior related experiments. We also integrated differentially expressed miRNAs to their target genes and categorized target genes for biological processes involved in the pathogenesis of DR.

## Main text

### Study subjects

A total of 170 patients, divided in three groups (60 healthy controls, 48 DM without DR patients and 62 DR patients) participated in this study and Additional file 1: Table S1 shows some clinicopathological characteristics of the recruited subjects. Universidade Estadual de Santa Cruz, Ilhéus, Bahia, Brazil Ethics Commitee approved the written consent that was taken from all the participants. The subjects were identified and classified by certified ophthalmologist that conducted fundus fluorescein angiography at CENOE (Clinica Especializada de Olhos, Ilhéus, Bahia, Brazil).

According to the guidelines from Global Diabetic Retinopathy Project Group [27], DR was diagnosed after routine fundus examination and fundus fluorescence angiogryphy examination. Patients with DM suffering from any form of hemangioma, small bleeding points, formation of new blood vessels, vitreous hemorrhage or secondary retinal detachment in the retina were classified as DR patients. Patients with diabetic ketosis, atherosclerotic disease and cardiac arrhythmias, trauma surgery, acute or chronic infection, hepatic disease and other endocrine metabolic diseases were excluded.

### Blood samples, RNA isolation and cDNA synthesis

Venous blood samples (5 mL) were collected from each donor in BD vacutainers dipotassium EDTA anticoagulant. Plasma fraction was separated by centrifugation. Plasma sample of 300 µL was mixed with 900 µL Trizol LS (Invitrogen) and RNA isolation was performed according to the manufacturer’s instructions. A NanoDrop 1000 (Thermo Scientific) was used to measure RNA concentration. Only RNA samples with a 260/280 ratio of ≥ 1.8 were included. 500 ng of total RNA was reverse transcribed using miR-specific primers and Taqman miRNA Reverse Transcription Kit (Applied Biosystem) in a scaled down volume of 15 µL RT reaction, according to the manufacturer’s instructions [28].

### Quantitative real-time PCR

Taqman MicroRNA assays (Applied Biosystems) and a QuantStudio3 Instrument (ThermoFisher Scientific) were used to measure expression levels of individual miRNAs by RT-qPCR. RT-qPCR amplification mixtures contained 20 ƞg template cDNA, 10 µL Taqman master mix (Applied Biosystems) and probes for MiR-320a (Assay ID: 002277), MiR-342-3p (Assay ID: 002260), MiR-155 (Assay ID: 002287), MiR-99a (Assay ID: 000435), MiR-29a (Assay ID: 002112) and MiR-27b (Assay ID: 000409) in a final volume of 20 µL. The PCR conditions were: incubation for 10 min at 95 °C, followed by 40 cycles of 10 s at 95 °C and 1 min at 60 °C. The Ct values for RT-qPCR were determined using the QuantStudio™ Design & Analysis Software (Applied Biosystems) and the single-threshold method. PCR reactions were performed in a duplicate and experiments with coefficients of variation greater than 5% or that displayed unusual amplification curves were excluded from further analysis. A no-template control (NTC) and no reverse transcription controls (No-RT) were also included. The mean cycle threshold (Ct) values from duplicate measurements were used to calculate expression of target gene, with normalization to an internal control miR-328-3p (Assay ID: 000543), which might be considered steady internal reference gene in expression studies on DR plasma samples [28], using 2 ^− ΔCt^ formula [29–31] and present as fold change.

### Computational prediction of potential miRNA targets

Target genes were predicted using miRWalk3.0 (http://mirwalk.umm.uni-heidelberg.de/). The miRWalk platform is based on predicted mRNA targets and integrates the predicted targets from various prediction tools: miRDB, TargetScan and miRTarbase. We setted filter for this tool with minimum score of 0.85. The miR targeting data and target gene interaction data were imported to Cytoscape, which was used to visualize and merge networks. Top ranked predicted genes were run through Ontology Genes (http://geneontology.org/) to perform enrichment analysis on gene sets and classification system to identify biological processes and reactome pathways associated with DR.

### Statistical analysis

Parametric data of all three groups were analyzed using one-way ANOVA with Tukey’s post hoc. All data were analyzed using the Prism 5.01 computer software (GraphPad, San Diego, CA, USA). Statistical differences were considered to be significant at *p *< 0.05.

### Results

#### Demographic and clinical profile of study subjects

The clinical characteristics of the patients are shown in Additional file 1: Table S1. Briefly, there were no significant differences in age and body mass index (BMI) between the three groups patients. Patients with DR were more often male and had a longer duration of diabetes compared to patients without DR. Moreover, daily insulin use was more frequent among patients with DR than in those without this complication.

#### Comparison of miRNA levels between study groups

**Fig. 1:**
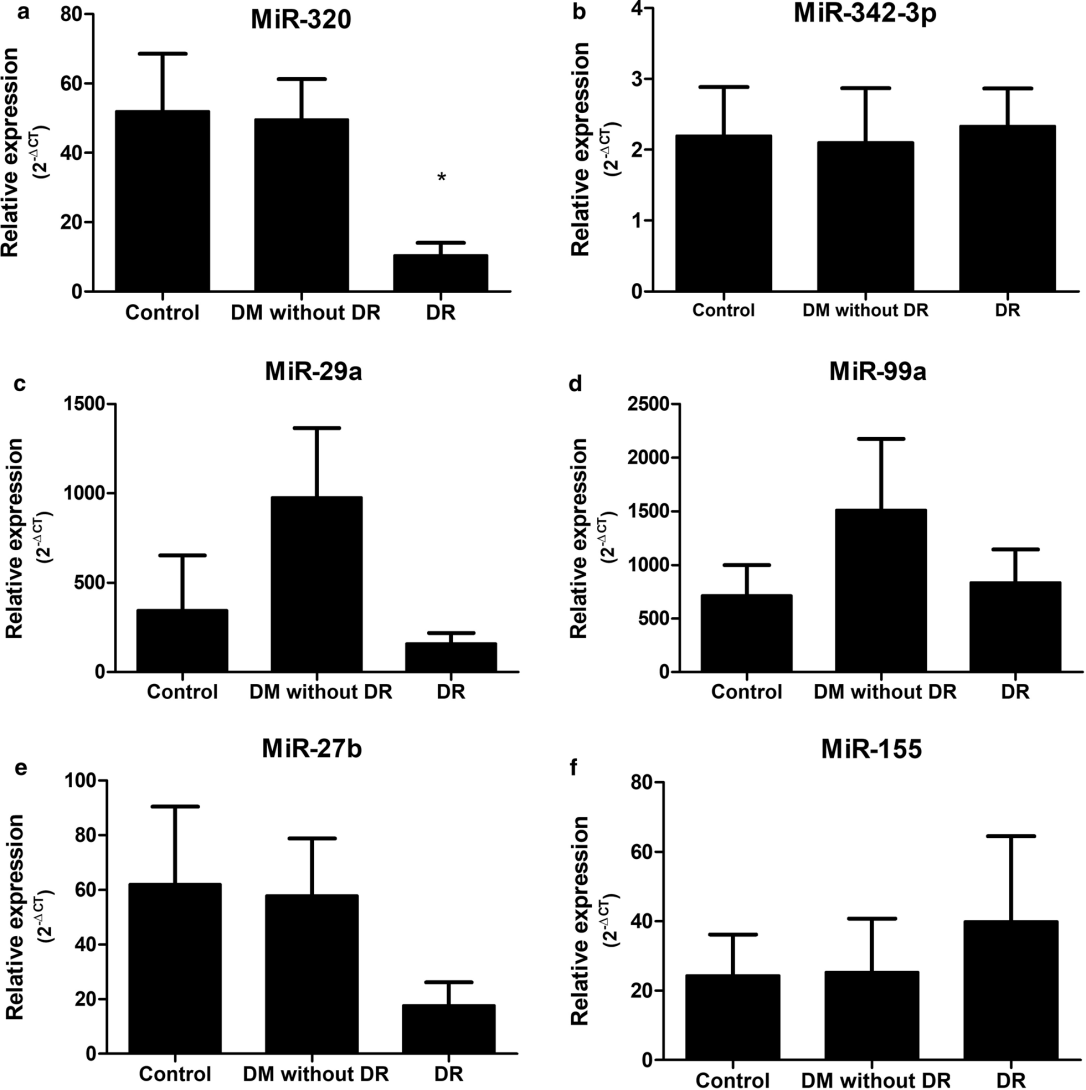
Circulating levels of miR-320a (**a**), miR-342-3p (**b**), MiR-29a (**c**), MiR-99a (**d**), MiR-27b (**e**) and MiR-155 (**f**) in healthy control subjects, diabetic patients without DR and diabetic patients with DR, evaluated by Taqman real-time PCR (arbitrary units). Data are represented graphically as the mean ± SEM of 48 to 62 subjects/group. *< 0.0001

Expression levels of MiR-320a, MiR-342-3p, MiR-155, MiR-99a, MiR-29a and MiR-27b were analyzed and, as shown in Fig. 1a, qRT-PCR analysis showed that circulating plasma level of miR-320a was profoundly downregulated in patients with DR compared to healthy subjects and DM patients (< 0.0001). Patients with DR had approximately five-fold lower levels of miR-320a in comparison to healthy subjects and DM patients without DR, which are not significantly different between them. No significant differences were observed for MiR-342-3p, MiR-155, MiR-99a, MiR-29a and MiR-27b expression (Fig. 1b–f).

#### Target gene prediction of miR-320a

**Fig. 2:**
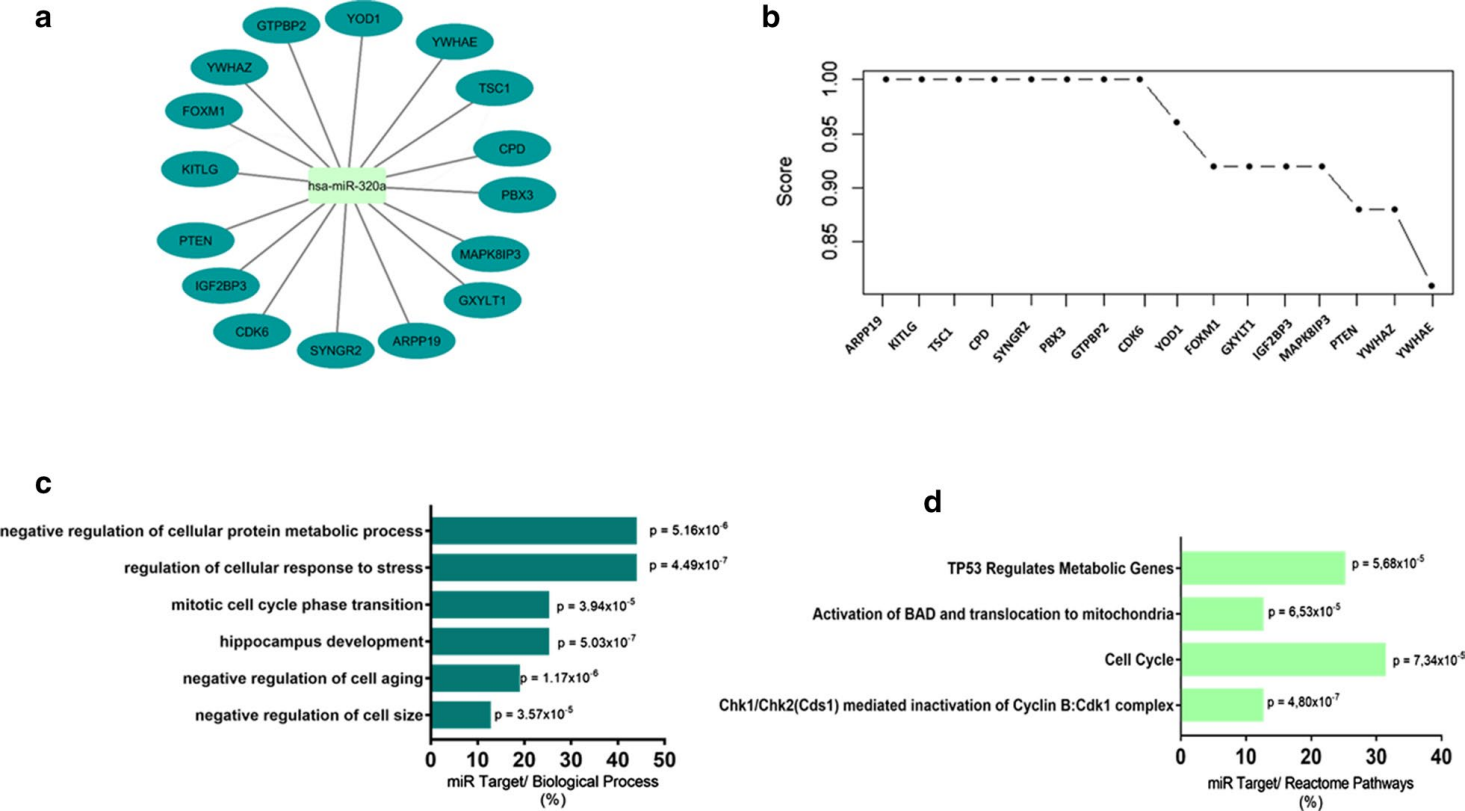
Target gene prediction with biology process and reactome pathways of miR-320a. **a** Interaction networks of miR-320 and target genes, based on analysis using miRWalk and **b** correlation between level significance by score. For this genes **c** Biological processes and **d** Reactome pathways with p-values were determined

As the miR-320a presented low expression in DR patients, with significant difference compared to the control group, we performed the in silico prediction to identify target genes possibly modulated by this miRNA in patients with DR. Based on analysis using miRWalk (integrating the miRDB, TargetScan and MiRTarbase), we observe that 16 genes are modulated (Fig. 2a) and were organized according to the significance presented by score in miRWAlk (Fig. 2b). From the analysis of functional enrichment, 6 significant biological processes (Fig. 2c) and 4 Reactome pathways (Fig. 2d) were found, which may indicate this miRNA involvement in important genes modulation in DR. The genes related to biological processes and reactome pathways are listed in Table 1.

**Table 1:** Biologic Process and reactome pathways for MiR-320a target prediction

Biology process	Acession	Genes
Negative regulation of cell size	GO:0045792	PTEN, TSC1
Negative regulation of cell aging	GO:0090344	PTEN, CDK6, FOXM1
Hippocampus development	GO:0021766	YWHAE, PTEN, TSC1, CDK6
Mitotic cell cycle phase transition	GO:0044772	YWHAE, CDK6, FOXM1, ARPP19
Regulation of cellular response to stress	GO:0080135	YWHAE, PTEN, CDK6, FOXM1, YOD1, MAPK8IP3, TSC1
Negative regulation of cellular protein metabolic process	GO:0032269	YWHAE, PTEN, ARPP19, YOD1, FOXM1, IGF2BP3, TSC1
Reactome pathways		
Chk1/Chk2(Cds1) mediated inactivation of Cyclin B:Cdk1 complex	R-HSA-75035.4	YWHAE, YWHAZ
Cell cycle	R-HSA-1640170.3	YWHAE, CD6, ARPP19, FOXM1, YWHAZ
Activation of BAD and translocation to mitochondria	R-HSA-111447.2	YWHAE, YWHAZ
Tp53 regulates metabolic genes	R-HSA-5628897.4	YWHAE, YWHAZ, PTEN, TSC1

## Discussion

Non-invasive and reliable biomarkers are needed to predict the risk of developing DM and its complications. Several researches have been focused on searching molecules involved in the pathogenic mechanisms at the basis of the development of DR [32, 33]. Circulating miRNAs have been largely addressed and investigated as non-invasive potential biomarkers in several diseases, including metabolic disorders [34]. Our experiment initially discovered that the levels of miR-320a are significantly down-regulated in the DR group comparing with those in the DM without DR and healthy control group.

Some studies have focused on finding an association between altered expression of MiR-320a and DM and DR. MiR-320 was found to regulate IGF-1 and IGF1R69 expression, playing a key role in developing insulin resistance in adipose tissues and endothelial cells [35]. Ling et al. also found that miR-320 augments insulin sensitivity in adipocyte in the insulin resistant condition, targeting PI3-Kp85 during the development of insulin resistance in adipocytes [36]. However, a few studies have focused on analyzing circulating miR-320a expression and the role of this miRNA and its targets in DR remains still unknown.

In the current study, several MiR-320a target genes were identified and top-ranking genes were ARPP19, KITLG, TSC1, CPD, SYGNR2, PBX3, GTPBP2 and CDK6. Among these genes the TSC1 and CDK6 are reported in the literature as important in DM. The *TSC1* negatively regulates mammalian target of rapamycin complex 1 (mTORC). TSC–mTOR pathway may result in the development of metabolic diseases and DM complications [37]. Curiously, the CDK6 gene was reported as an inductor of pancreatic β-cell replication and human islets proliferation by Fiaschi-taesch et al. [38] The CDK6 is still suppressed indirectly by upregulation miRNAs in DM and complications of the disease [39, 40].

However, in this study a negative reduction in MiR-320a was identified in patients with DR compared to the control group and the group with DM without DR, what could mean an increase in the CDK6 gene expression [41]. The low MiR-320a expression would lead to a high expression of CDK6, due to a dysregulation in the cell cycle mechanism, since this pathology would cause vascular and cellular changes [2].

Using Gene Ontology (GO) classification system, genes were categorized into several biological processes that are critical in the course of DR, including regulation of cellular response to stress [42, 43], negative regulation of cellular protein metabolism process [44], mitotic cell cycle phase transition [45, 46]. In conclusion, we found a five-fold downregulation of miR-320a in the plasma of patients with DR. Integrated genes were identified for this miRNA and we divided top-ranked genes into biological process that are critical for DR based on total target scores. Despite our results, our data has several limitations. Our experimental context does not allow to infer about the mechanism by which DM duration has a different effect on the circulating miRNAs expression profile in different groups, because we could investigate the expression of only some specific microRNAs. Besides this, we had a small sample size that could make more difficult for us to identify significant relationships from our data. Additionally, the analysis of a bigger number of miRNAs expression could be much more informative about new candidates for DR biomarker use, but we were able to evaluate only six candidate microRNAs that were selected from previously published data and based on prior related experiments. Our findings suggest that miR-320a may have a role in the pathogenesis of DR, but other future studies are needed to investigate if this circulating microRNA has clinical importance or if it would permit an accurate identification of risk factors or prevention of events.

## Limitation of the study

- The study was conducted only in a single hospital.
- Despite computer-based prediction methods are valuable in preliminary identification of miRNA target genes, inherent limitations should be considered when applying the results of these searches to experimental validation.

## Supplementary information

**Additional file 1: Table S1.** Clinical characteristics of the patients.

## Data Availability

The datasets used and/or analyzed during the current study are available from the corresponding author on reasonable request.

## Abreviations

MiRs: MicroRNAs
DM: Diabetes melitus
DR: Diabetic retinopathy
EDTA: Ethylenediaminetetraacetic acid
RNA: Ribonucleic acid
DNA: Desoxyribonucleic acid
cDNA: Complementary DNA
PCR: Polymerase Chain Reaction
qRT-PCR: Quantitative reverse transcribed PCR
NTC: no-template control
No-RT: No reverse transcription
ANOVA: Analysis of variance
