# Supplementary material for: Downregulation of circulating miR-320a and target gene prediction in patients with diabetic retinopathy
Source: BMC Res Notes. 2020 Mar 16;13:155. doi: 10.1186/s13104-020-05001-9 (PMC7077016; doi:10.1186/s13104-020-05001-9)
Supplement: Supplementary file 1 — Additional file 1: Table S1. Clinical characteristics of the patients. [file 13104_2020_5001_MOESM1_ESM.docx]

| **Characteristics** | **Control Subjects** | **NDR subjects** | **DR subjects** | **P value** |
| --- | --- | --- | --- | --- |
| n | 60 | 48 | 62 |  |
| Age (years) | 54.2±13.3 | 58.6±8.95 | 58.3±6 | 0.09 |
| Gender (male/female) | 33/27 | 11/37 | 29/33 | 0.02* |
| Course of disease (years) | - | 5±5.2 | 17±10.3 | <0.0001* |
| Alcohol consumption | 28 | 16 | 18 | 0.11 |
| Current Smoker | 10 | 19 | 23 | 0.01* |
| Kidney disease | 1 | - | 1 | 0.67 |
| Cardiovascular disease | 1 | 1 | 3 | 0.53 |
| BMI (kg/m^2^) | 27±3.5 | 29±5.6 | 27±5.4 | 0.19 |
|  |  |  |  |  |
| **Medication** |  |  |  |  |
| Biguanides (%) |  | 50% | 54% |  |
| Sulfonylureas (%) |  | 25% | 45.1% |  |
| SGLT2 Inhibitors (%) |  | 2% | 0 |  |
| Insuline |  | 10% | 56.4% |  |

**Table 1.** Clinical characteristics of the patients.

* P<0.05
